# Editorial: Community series in tumor ablation and immunity, volume II

**DOI:** 10.3389/fimmu.2024.1457368

**Published:** 2024-07-08

**Authors:** Yueyong Xiao

**Affiliations:** Department of Radiology, First Medical Center, Chinese PLA General Hospital, Beijing, China

**Keywords:** tumor, ablation, immunity, synergetic effect, immunotherapy

Tumor ablation therapy is to locally destroy tumors by using advanced medical equipment to deliver different physical or chemical energies to solid tumors through a medium such as an ablation probe. Ablation therapy has been widely applied in clinical practice due to its minimal invasive, without resection of tissues or organs, high local therapeutic effects, patient fast recovery and can be used repeatdly. For early-stage patients, its efficacy can even be comparable to that of surgical resection, and it is especially suitable for patients who are unable to undergo surgical procedures for various reasons. Ablation therapy has long been regarded as a kind of clinical local treatment, and combined with the overall systemic treatment of the patient has been always a project of clinical quest and exploration. Along with the popularity of applying of ablation therapy, it has been found that the commonly used cryoablation, radiofrequency ablation, microwave ablation, and irreversible electroporation ablation can cause changes in systemic immune function and enhance the body’s anti-tumor immune response while inactivating the tumor cells *in situ*. In order to further focus on the relationship between tumor ablation and immunity, we have co-worked with the high-impact frontiers journal to launch a Research Topic on “Tumor Ablation and Immunity” since 2022, which has gained great attention from international scholars and a large number of high-level studies had been published after rigorous peer review. The Research Topic was divided into two issues and totally 41 papers were published. In this editorial, we will summarize the latest research results and opinions in the field of tumor ablation and immunity through the papers published in this Research Topic, and illustrate the promising future of the synergistic anti-tumor effects of tumor ablation and immunity.

De Miranda et al. published a review on the combination of cryoablation and immune checkpoint inhibitors in high-risk triple-negative breast cancer. This mini-review analyzed the pros and cons of cryoablation for the treatment of breast cancer and suggests that cryoablation may produce reliable abscopal effects. The authors not only analyzed the immune effects of cryo, but also described the basic principles of cryoablation and ICI therapy and discussed strategies aimed at improving this approach for the benefit of patients and considerations in current clinical trials. In addition, the authors discussed the effectiveness of low/ultra-low doses of ICI in combination with cryoablation to minimize the side effects of ICI while achieving optimal efficacy. In contrast, local delivery of ICI reduces the frequency and severity of irAEs and also improves the efficacy of the anti-tumor response. Subsequent prospective clinical trials are needed to demonstrate the clinical benefit of this combination therapy strategy in patients with triple-negative breast cancer.

Liu et al.‘s study also focused on the combination of cryoablation and immune checkpoint inhibitors. They described the immune mechanism of cryoablation and concentrated on the theoretical basis and research progress of the combination of cryoablation and immune checkpoint inhibitors in the treatment of cancer. Tumor antigens released by cryoablation are not sufficient to induce a significant anti-tumor immune response; however, ICI can block PD-1 binding to PD-L1 and CTLA-4 binding to CD80/CD86, which releases the “braking” mechanism of the co-stimulatory signaling pathway and restores the activation and proliferation of T cells. Tumor-specific T cells synergistically activated by cryoablation and ICI therapy have the ability to recognize and destroy local residual tumors as well as distant macroscopic and microscopic metastases. Findings from numerous preclinical and clinical trials collectively support the potential of cryoablation combined with ICI therapy for the treatment of a variety of solid tumors, demonstrating favorable safety and efficacy profiles. The authors also proposed that depletion of Tregs will be considered as a potential approach for enhancing the efficacy of cryoimmunomodulation, providing new perspectives on tumor therapy.

Ye and Sang published a review of potential biomarkers for predicting immune responses and outcomes in lung cancer patients undergoing thermal ablation. They provided a comprehensive overview of the current state of research on potential biomarkers for predicting immune responses and outcomes in lung cancer patients undergoing thermal ablation, including their potential roles in the management of lung cancer as well as the challenges and future directions. Biomarkers have become valuable tools for lung cancer diagnosis, prognosis, and treatment selection, providing important insights into the molecular mechanisms behind the disease. The identification and validation of biomarkers has dramatically changed the management of lung cancer in several ways, including diagnosis, prognosis, and treatment selection. Combining multiple biomarkers can improve the accuracy of predicting outcomes for lung cancer patients undergoing thermal ablation and help clinicians tailor individualized treatments to each patient’s unique disease biology.

The study by Hadzialjevic et al. focused on the combined use of electrochemotherapy and immunotherapy. Electrochemotherapy uses electrical pulses that increase the permeability of the cell membrane of the target lesion, which enhances the delivery of hypo-osmotic cytotoxic drugs to the cell, leading to cell death. Electrochemotherapy acts as an *in situ* vaccination by inducing immunogenic cell death, which in turn provides a theoretical basis for integration with immunotherapy. The combination of electrochemotherapy and ICI therapy has been demonstrated in animal experiments and clinical studies, and is currently being used in the clinical management of melanoma, breast cancer, cutaneous squamous cell carcinoma, and hepatocellular carcinoma. Subsequently, more preclinical and translational studies are needed to better explain the potential mechanisms of the combination therapy and validate its efficacy and safety.

Imran et al. published an animal study of irreversible electroporation for the treatment of pancreatic cancer. In this study, the authors found that irreversible electroporation induced alterations in the local tumor microenvironment, resulting in a decrease in myeloid-derived suppressor cell (MDSC) and regulatory T-cell populations, as well as an increase in cytotoxic T-lymphocytes and neutrophils, which in turn altered the immunosuppressive tumor microenvironment. The authors also found that IRE enhanced the IFN-γ signaling pathway, leading to the upregulation of the PD-L1 checkpoint in pancreatic cancer cells, which provides a theoretical basis for the synergistic antitumor effects of IRE and PD-L1 inhibitors. IRE may promote the expression of PD-L1 in tumor cells and provide additional targets for PD-L1 inhibitors, and the combination of the two reversed immunosuppression in pancreatic cancer. The synergistic antitumor effects of IRE and ICI need to be verified in more preclinical experiments and clinical trials in the future.

Meng and Wei from the team of the Chinese PLA General Hospital has summarized and published their nearly 20 years of basic and clinical applied researches on chemo-immunoabaltion. While inactivating the tumor cells through intratumoral injection of ablating agents to the solid tumors, the microenvironment of the tumor mesenchyme be completely changed, and the long-term residence of the ablating agents in the tumor body allows for the complete inactivation of the tumor cells. These techniques are particularly suitable for tumors growing in special regions where physical ablation is not possible. Further basic research results, such as improvement of local ablating agents, slow release of drugs, and combined PDL1 intratumor injection, are also being summarized and are expected to be published.

In conclusion, in the Volume II of the Research Topic on “Tumor Ablation and Immunity”, we were surprised to find that more and more clinicians and scientists in the field are focusing on the immune effects induced by tumor ablation, and have discovered that different immunotherapies may have an additive effect on the immune response to tumor ablation, and that the combination of the two may lead to local inactivation of tumors and comprehensive systemic therapy. In the future, more evidence-based medical evidence and research will further explore the synergistic potential of tumor ablation and immunotherapy, bringing new breakthroughs in integrated tumor therapy.

## Author contributions

YX: Writing – original draft, Writing – review & editing.

